# Comparative Genomics of *Rhodococcus equi* Virulence Plasmids Indicates Host-Driven Evolution of the *vap* Pathogenicity Island

**DOI:** 10.1093/gbe/evx057

**Published:** 2017-05-01

**Authors:** Iain MacArthur, Elisa Anastasi, Sonsiray Alvarez, Mariela Scortti, José A. Vázquez-Boland

**Affiliations:** 1 Division of Infection and Immunity, The Roslin Institute, University of Edinburgh, Edinburgh, United Kingdom; 2 Edinburgh Medical School (Biomedical Sciences), University of Edinburgh, Edinburgh, United Kingdom; 3 Centre for Immunity, Infection and Evolution, University of Edinburgh, Edinburgh, United Kingdom

**Keywords:** *Rhodococcus equi*, pangenome analysis, comparative genomics, host-associated virulence plasmids, host tropism

## Abstract

The conjugative virulence plasmid is a key component of the *Rhodococcus equi* accessory genome essential for pathogenesis. Three host-associated virulence plasmid types have been identified: the equine pVAPA and porcine pVAPB circular variants, and the linear pVAPN found in bovine (ruminant) isolates. We recently characterized the *R. equi* pangenome (Anastasi E, et al. 2016. Pangenome and phylogenomic analysis of the pathogenic actinobacterium *Rhodococcus equi*. Genome Biol Evol. 8:3140–3148.) and we report here the comparative analysis of the virulence plasmid genomes. Plasmids within each host-associated type were highly similar despite their diverse origins. Variation was accounted for by scattered single nucleotide polymorphisms and short nucleotide indels, while larger indels—mostly in the plasticity region near the *vap* pathogencity island (PAI)—defined plasmid genomic subtypes. Only one of the plasmids analyzed, of pVAPN type, was exceptionally divergent due to accumulation of indels in the housekeeping backbone. Each host-associated plasmid type carried a unique PAI differing in *vap* gene complement, suggesting animal host-specific evolution of the *vap* multigene family. Complete conservation of the *vap* PAI was observed within each host-associated plasmid type. Both diversity of host-associated plasmid types and clonality of specific chromosomal-plasmid genomic type combinations were observed within the same *R. equi* phylogenomic subclade. Our data indicate that the overall strong conservation of the *R. equi* host-associated virulence plasmids is the combined result of host-driven selection, lateral transfer between strains, and geographical spread due to international livestock exchanges.

## Introduction

*Rhodococcus equi* is a species of pathogenic *Actinobacteria* that causes pulmonary and extrapulmonary pyogranulomatous infections in animals and people (Prescott 1991). As is typical in the *Rhodoccus* genus, *R. equi* niche specialization is plasmid determined. The plasmid-encoded functions are mainly catabolic in the environmental rhodococci (Larkin et al. 2010) whereas in *R. equi* they promote animal host colonization (von Bargen and Haas 2009; Vázquez-Boland et al. 2013). The *R. equi* virulence plasmid carries a horizontally acquired pathogenicity island (PAI), the *vap* PAI, that confers the ability to parasitize macrophages (Hondalus and Mosser 1994; Giguère et al. 1999; Takai et al. 2000). The *vap* PAI contains a set of homologous genes encoding small-secreted virulence-associated proteins (Vap), one of which (exemplified by VapA from the equine-type plasmid, see below) is essential for pathogenesis (Coulson et al. 2010; Valero-Rello et al. 2015). It also contains a number of non-*vap* genes, notably the *vir* operon harboring two regulators (*virR* and *virS*) that activate the expression of the PAI (Byrne et al. 2007). A number of *R. equi* chromosomal genes appear to have been coopted to serve a function in virulence in a *vap* PAI-coregulated network, presumably under the control of *virR* and *virS* (Letek et al. 2010; Coulson et al. 2015).

The *R. equi* virulence plasmid appears to have a second key function as a host tropism determinant. Three host-specific virulence plasmid types have been identified, the circular pVAPA and pVAPB, associated, respectively, with equine and porcine isolates (Letek et al. 2008), and the recently characterized linear plasmid pVAPN associated with bovine isolates (Valero-Rello et al. 2015). The three plasmid types are found in human isolates (Ocampo-Sosa et al. 2007), consistent with people being opportunistic hosts for *R. equi* and a zoonotic origin of human infection.

We recently carried out an analysis of the *R. equi* pangenome based on a panel of representative isolates from different sources (Anastasi et al. 2016). Taking advantage of the genomic and epidemiological diversity of the strains in that study, here we report a detailed comparative genomic analysis of their corresponding virulence plasmids. The data herein provide insight into the population genomics and evolution of the *R. equi* host-associated virulence plasmids, a key niche-adaptive component of the pathogen’s accessory genome.

## Materials and Methods

A total of 25 *R. equi* virulence plasmids of the three host-associated types (15 equine type pVAPA, four porcine type pVAPB, and six bovine type pVAPN) were analyzed in this study, including 20 new plasmid sequences and the reference plasmids of each type (pVAPA1037, pVAPB1593, and pVAPN1571) (Takai et al. 2000; Letek et al. 2008; Valero-Rello et al. 2015) (supplementary table S1, Supplementary Material online). The host strains were from different sources (equine, porcine, bovine, ovine, and human) and geographical origins (13 countries). De novo plasmid sequences were derived as single contigs from 101 bp paired-end Illumina HiSeq 2000 shotgun genomic sequences assembled and analyzed as described in Anastasi et al. (2016). Ambiguities were resolved by PCR mapping and sequencing of PCR products. Other specific methods are indicated where relevant. The *R. equi* virulence associated plasmids (pVAP) are designated according to a normalized nomenclature with the host-associated type A, B, or N as a suffix followed by the strain identifier (Letek et al. 2008; Valero-Rello et al. 2015).

## Results and Discussion

### pVAPA Plasmids

The first *R. equi* virulence plasmid type to be genomically characterized was the equine-associated pVAPA. Two examples, plasmids p33701 and p103 of 80.6 kb, from strains ATCC33701 and 103 isolated from foal lung in Canada, were initially sequenced and reported to be virtually identical (Takai et al. 2000). The p103 plasmid was resequenced in our laboratory from subculture 103S used to determine the complete genome of *R. equi*. This plasmid was designated pVAPA1037 and is the reference for the pVAPA type (Letek et al. 2008, 2010). A subculture of ATCC33701 kept in our collection as PAM1271 was sequenced in the *R. equi* pangenome study (Anastasi et al. 2016) and its plasmid, pVAPA1271, is analyzed here (supplementary table S1, Supplementary Material online). See Supplementary text S1, Supplementary Material online, for a comparison between the 103/103S and ATCC33701/PAM1271 plasmid sequences.

**Fig. 1.—:**
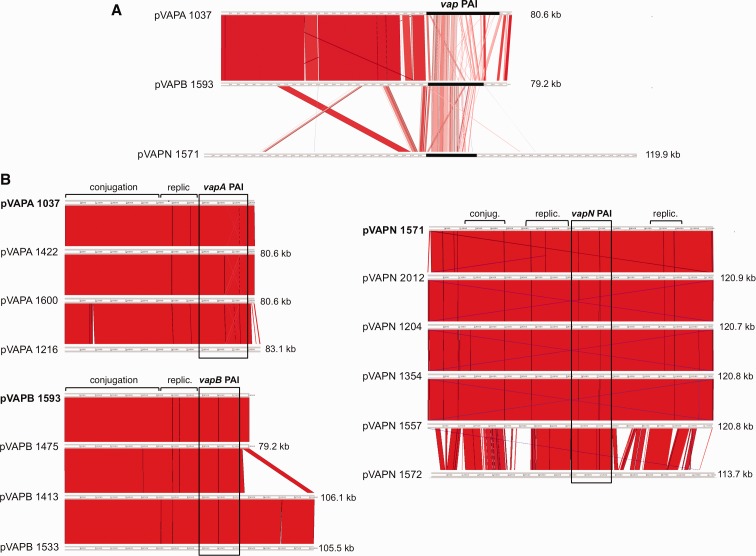
Genome comparisons of *R. equi* host-associated virulence plasmids. Nucleotide sequences aligned using Artemis Comparison Tool (ACT) (Carver 2005). Similar regions are joined by red stripes (blue when in reverse orientation), paler shade indicates decreasing identity. (*A*) Alignment of the reference sequences of the equine-type (pVAPA1037, GenBank accession number AM947677), porcine-type (pVAPB1593, GenBank accession number AM947676), and bovine (ruminant)-type (pVAPN1571, GenBank accession number KF439868) virulence plasmids. Blastn identity cutoff 70%, size cutoff 30. The position of the *vap* PAI is indicated; note in the pVAPA and pVAPB circular plasmids the lower degree of nucleotide sequence conservation of the *vap* PAI compared to the housekeeping backbone. A detailed description of the reference plasmids of each type can be found in Letek et al. (2008) and Valero-Rello et al. (2015). (*B*) Comparison of three representative new pVAPA plasmid sequences and all sequenced pVAPB and pVAPN plasmids to their corresponding reference plasmids (in bold). Blastn identity cutoff 98%. Main plasmid housekeeping modules and *vap* PAIs are indicated. Other 10 pVAPA plasmids not shown are** **>99% identical to pVAP1037. pVAPA1216 differs by two additional CDS between pVAPA_*0040* and _*0050* and the replacement of pVAPA_*0830* and _*840* by four new CDS (gene nomenclature according to Letek et al. [2008]). The latter indel is adjacent to the invertase/resolvase *invA* gene flanking the right end of the *vap* PAI in pVAPA/B’s plasticity region (Letek et al. 2008). The identical 17-gene insertion in pVAPB1413 and pVAP1533 is also at the right end of the *vapB* PAI adjacent to the *invA* gene; it encodes two transposases, a TnB transposition protein and a phage integrase, plus a bleomycin resistance determinant, an MFS transporter, and β-oxidation catabolic enzymes with closest homologs in other rhodococci. Most pVAPN plasmids (not at scale) are identical to the reference sequence except for two additional CDSs near the left telomere (Valero-Rello et al. 2015). Blue lines joining the termini indicate the telomeric inverted repeats.

Of the 13 new pVAPA sequences, 12 are identical to pVAPA1037 except for a few SNPs (maximum nine) and short 1–8 bp indels (fig. 1 and supplementary fig. S1, Supplementary Material online). The sequence variation occurs at a similar frequency across the plasmid backbone and PAI (2.34 × 10^−4^ and 3.02 × 10^−4^, respectively), likely reflecting normal genetic drift. pVAPA1037 is a “85 kb” type I plasmid, one of the major pVAPA restriction fragment length polymorphism (RFLP) subtypes among 12 described for the equine plasmids (Duquesne et al. 2010) (note that the RFLP size designation does not correspond to the actual plasmid size). The four subtypes within this RFLP group, I to IV, most likely represent sequence microvariants of the 80.6 kb pVAPA plasmid. The other equine plasmid, pVAPA1216 (83.1 kb) from a Mexican isolate, differed from pVAP1037 by a large number of SNPs and gene indels at two sites outside the *vapA* PAI (fig. 1 and supplementary fig. S1, Supplementary Material online). Its genetic structure is identical to that of pVAPAMBE116, of a different equine RFLP subtype (“87 kb” type I), previously sequenced from a foal isolate in France (Duquesne et al. 2010).

### pVAPB Plasmids

The 79.2 kb pVAPB1593 plasmid is the first and only sequenced example of the porcine-associated pVAPB type (Letek et al. 2008), also frequently found in human isolates (Takai et al. 1996, Makrai et al. 2002). pVAPB1593 shared with the equine pVAPA1037 the same housekeeping backbone but had a significantly divergent *vap* PAI (Letek et al. 2008). The common circular backbone was similar to that of the pREC1 plasmid from the alkane and methylbencene degrader, *Rhodococcus erythropolis* PR4 (Sekine et al. 2006). It was therefore clear that the pVAPA and pVAPB types were derived from a single original plasmid, a widespread rhodococcal extrachromosomal element that had acquired the *vap* PAI by horizontal transfer and in which this virulence locus evolved into two host species-specific variants (Letek et al. 2008; Vázquez-Boland et al. 2013). In addition to being smaller in size (15.9 vs. 21.5 kb for pVAPA; Valero-Rello et al. 2015) and having six instead of nine *vap* multigene family members, Vap polypeptide sequence variability, *vap* gene rearrangements and indels affecting adjacent genes accounted for the PAI differences between the A and B plasmid variants (Letek et al. 2008).

Of the three new pVAPB genomes, pVAPB1475 (porcine isolate, Hungary) has the same genetic structure as the reference pVAPB1593 sequence (human isolate, Spain; Letek et al. 2008) while pVAPB1413 and pVAPB1533 (porcine and human origin, respectively) are considerably larger (≈106 kb). The difference in size is due to a same ≈27 kb insertion immediately after the right end of the *vapB* PAI. This additional plasmid segment comprises various metabolic enzyme genes flanked by Tn*7*-like transposase and tyrosine recombinase genes (fig. 1). Insertion events in the plasticity region thus appear to contribute to the reported variability of pVAPB plasmids (for which at least 27 RFLP subtypes with sizes ranging from 79 to 100 kb have been reported; Makrai et al. 2008). Otherwise, both the *vapB* PAI and the housekeeping backbone are strongly conserved in the pVAPB plasmids (fig. 1).

### pVAPN Plasmids

Apart from carrying a copy of the *vap* PAI, the recently reported bovine-associated pVAPN type was completely unrelated to the circular pVAPA/B plasmids (Valero-Rello et al. 2015). Its backbone was an invertron-like conjugative replicon similar to the linear pNSL1 plasmid found in an environmental *Rhodococcus* sp. (strain NS1). This linear replicon acquired the *vap* locus by horizontal mobilization, presumably from a direct precursor of pVAPA. The pVAPN PAI again contained a specific complement of *vap* genes one of which, *vapN*, was identified as the “bovine” homolog of the equine *vapA* (and found to be also essential for virulence) (Valero-Rello et al. 2015).

Three of the four new pVAPN plasmids, each from different geographical origins and host sources (supplementary table S1, Supplementary Material online), are genomically identical to the previously sequenced pVAPN2012 (Valero-Rello et al 2015) (fig. 1). As seen for the pVAPA and pVAPB plasmids, they only differ in a number of SNPs and short (1–18 nt) indels. These plasmids are also virtually identical to the reference sequence pAPN1571 (bovine, Ireland; GenBank accession number KF439868), except for two additional ORFs encoding hypothetical proteins near one of the telomeric ends (Valero-Rello et al. 2015; fig. 1). The fourth plasmid, pVAPN1572 (bovine, Ireland), is considerably divergent (fig. 1). A total of 44 CDS from the reference sequence, mostly encoding hypothetical proteins, are not conserved in pVAPN1572. Among them, interestingly, pVAPN_0500 (*parA*), which is replaced (at the same location) by an unrelated gene encoding a ParA protein similar (26% identity) to that encoded by pVAPA/B. This further supports the notion that the *rep*-*parA* determinant of the *Rhodococcus* plasmids (often identified as horizontal gene transfer [HGT] DNA) is part of a highly plastic, horizontally exchangeable plasmid region (Letek et al. 2008; Valero-Rello et al. 2015). In addition, pVAPN1572 encodes a ParB/SpoOJ plasmid partitioning protein, which is not present in the other pVAPN plasmids (Valero-Rello et al. 2015). pVAPN1572 in turn possesses 23 unique genes, again mostly encoding hypothetical proteins. The significant divergence of pVAPN1572 is due to the accumulation of a large number of indels in the accessory plasmid backbone. Despite the extensive differences, the key housekeeping modules involved in conjugation (including the TraB plasmid translocase pVAPN_0320; Valero-Rello 2015) and replication are strongly conserved (fig. 1).

Of note, one of the pVAPN plasmids (of the pVAPN2012-type) belongs to an ovine isolate. Other sheep and goat isolates kept in our *R. equi* collection were also found to carry a pVAPN plasmid, suggesting that this type is generally associated with ruminants.

### Conservation of Host-Associated *vap* PAIs

Each host-associated plasmid type possesses a perfectly conserved type-specific *vap* PAI with a unique *vap* gene complement (fig. 1 and supplementary fig. S2, Supplementary Material online). There are only a small number of SNPs and indels generally in intergenic regions or representing synonymous nucleotide substitutions, hence the PAI product identity is 100% within each type, including the otherwise highly variable Vap multigene family proteins (supplementary table S2, Supplementary Material online). A number of non-*vap* genes are conserved across the A-, B-, and N-type PAIs. These include (from left to right; see supplementary fig. S2, Supplementary Material online) *cgf* encoding a CopG family transcriptional regulator (Valero-Rello et al. 2015); *lsr2* encoding a homolog of the mycobacterial nucleoid-associated protein Lsr2; a two-gene locus (*pVAPA_0460-470*) in the opposite orientation encoding a hypothetical protein (pseudogene in pVAPA) and an *S*-adenosylmethionine (SAM)-dependent methyltransferase with a potential regulatory role; the five-gene *vir* operon including *virR* (aka *orf4*, LysR-type transcriptional regulator), *icgA* (major facilitator superfamily transporter; Wang et al. 2014), a *vap* gene (VapH, -J, -P), a conserved hypothetical gene, and *virS* (aka *orf8*, two-component response regulator; Byrne et al. 2007); and *vcgB* encoding a conserved hypothetical protein (Miranda-Casoluengo et al. 2011). The cross-type homologous non-*vap* gene products show an overall high degree of amino acid sequence conservation, especially those encoded by *cgf*, *lsr2*, and the *vir* operon (≈90% identity) (supplementary table S2, Supplementary Material online). 

The level of similarity is much lower among the Vap proteins across the three plasmid types (≈60–70% identity range), if the coding gene is not lost or corrupted. Indeed, apart from a few small hypothetical genes that are not conserved, the main differences between the three PAI types lie in the *vap* genes. Our previous phylogenetic analyses determined that the *vap* multigene family members present in pVAPA, pVAPB, and pVAPN all descend from a common PAI ancestor that carried seven *vap* genes (Valero-Rello et al. 2015). This precursor *vap* gene set underwent divergent evolution in the three host-associated types, involving *vap* gene rearrangements, duplications, gene loss and decay processes, and sequence diversification in PAI type-specific allelic variants (Letek et al. 2008; Valero-Rello et al. 2015). This is illustrated by the Vap encoded in the strongly conserved *vir* operon, which shows a much higher degree of divergence compared to the products of the surrounding *vir* operon genes (supplementary table S2, Supplementary Material online).

### Distribution of Host-Associated Plasmids in *R. equi* Population Structure

**Fig. 2.—:**
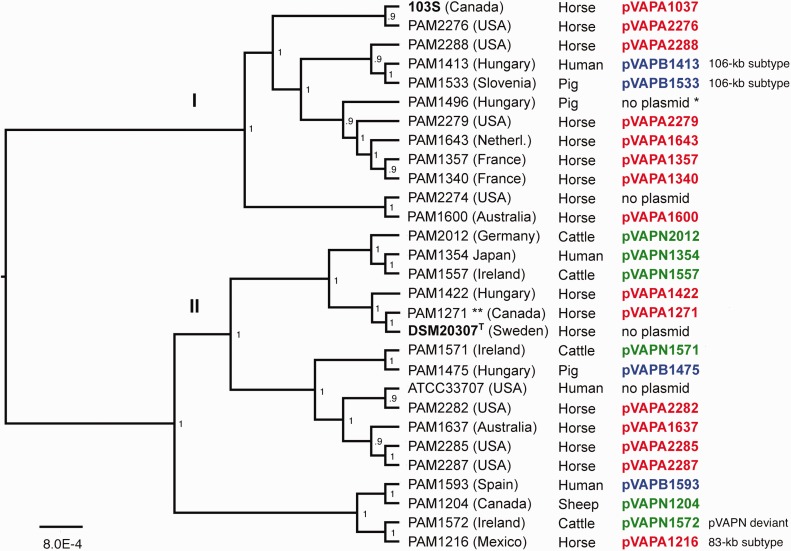
Distribution of host-associated virulence plasmid types in *R. equi* population structure. Maximum likelihood core-genome tree inferred from 29 *R. equi* genome sequences (Anastasi et al. 2016) using RealPhy program (Bertels et al. 2014). Midpoint rooted and visualized with FigTree v1.4.2. with branch length proportional to the tips under the node; scale bar refers to overall distance and indicates substitutions per site. Nodes indicate bootstrap support from 500 replicates. Tip labels show strain designation, geographical origin, host source and plasmid name (color-coded according to host-associated plasmid type: pVAPA, red; pVAPB, blue; pVAPN, green). In bold, the reference (complete) genome strain 103S (Letek et al. 2010) and the type strain of the species (DSM 20307^T^). *, isolate PAM1496 originally positive to pVAPB but plasmid lost in sequenced clone. **, PAM1271 is a subculture of strain ATCC33701.

Collating the distribution of the virulence plasmids in a *R. equi* phylogenomic tree shows that different host-associated plasmid types can be found within a specific terminal subclade, and that the three plasmid types can be found within a same chromosomal phyletic line (fig. 2). Clustering of specific plasmid types can be observed at the top of the tree (e.g., pVAPN in the terminal clade formed by strains PAM2012, -1354, and -1557), likely reflecting (plasmid-directed) clonal grouping of niche (host)-restricted genomotypes. Supporting this, the two pVAPB plasmids of the 106 kb type (pVAPB1413 and -1533) are found within a same terminal bifurcation of the core genome tree. The pVAPN type seems to be restricted to lineage II strains (Anastasi et al. 2016), but no specific conclusions can be drawn because of the smaller number of strains from this phylogenomic division (fig. 2). Overall, although the genome sample analyzed is relatively small and horse isolates predominate, our findings are indicative of active exchange of host-associated virulence plasmids types across the *R. equi* population.

Four strains, DSM20307^T^ (equine isolate from the time of first description of *R. equi*; Magnusson 1923), ATCC33707 (human), PAM2274 (equine), and PAM1496 (porcine), were devoid of virulence plasmid (fig. 2). The proportion of “plasmidless” *R. equi* is generally small among infection-related isolates (5–14% of equine strains [Ocampo-Sosa et al. 2007], 0% of bovine isolates in our collection) but significant in isolates from nonpathological sources (≈30% of porcine strains, most from healthy pig carriers; ≈40–50% of environmental isolates [Ocampo-Sosa et al. 2007; Duquesne et al. 2010; our unpublished observations]). Spontaneous plasmid curing can be observed experimentally during in vitro subculturing of *R. equi*, presumably due to fitness cost (Takai et al. 1994; Gonzalez-Iglesias et al. 2014; Valero-Rello et al. 2015). These observations are consistent with virulence plasmid maintenance depending on a balance between host-driven positive selection and purifying selection outside the host. The spread of the four plasmidless strains across different phylogenomic lineages/divisions suggests that the ability to lose the virulence plasmid is independent of the *R. equi* genetic background.

## Conclusions

Despite the geographic diversity of the strains analyzed, the virulence plasmids of each of the three host-adapted types are generally strongly conserved. With the exception of the deviant pVAPN1572 plasmid found in a bovine isolate, the variation is limited to a number of SNPs, short bp indels and, in some cases, larger indels involving from two to up to 24 genes. The latter define plasmid genomic subtypes within each host-associated type. The microvariation is largely scattered across the plasmids’ sequence whereas the larger indels concentrate in the plasticity region where niche-adaptive HGT DNA insertions take place, including the *vap* PAI itself (Letek et al. 2008; Valero-Rello et al. 2015).

We previously found a lack of correlation between *R. equi* genotypes and virulence plasmid types (Vázquez-Boland et al. 2008; Anastasi et al. 2016; our unpublished observations) and the genomic analysis of the plasmids confirms this. Our observations are consistent with a scenario of saprophyte-parasite transitions underpinned by the dynamic loss, reacquisition and conjugal transfer of host-specific virulence plasmids (with concomitant host jumps). Lateral exchange and worldwide spread due to international livestock trade (Anastasi et al. 2016), also suggested by the genomic analysis of the plasmids, is another factor likely accounting for the observed strong conservation of the host-associated virulence plasmids.

The analyses also show that strains with identical chromosomal genomotype and host-associated plasmid genomic subtype can be isolated from both the corresponding animal host and people, further supporting that human *R. equi* infections are zoonotic (Ocampo-Sosa, et al. 2007; Anastasi et al. 2016).

The pVAPA, pVAPB, and pVAPN plasmids possess each a unique *vap* PAI, consistent with a common ancestral *vap* locus undergoing divergent evolution in each host-associated plasmid type (Valero-Rello et al. 2015). The conservation of the DNA mobility genes flanking the *vap* PAI (*resA* and *invA* in pVAPA/B, *tniA*, and *tniQ* in pVAPN) and of the pseudogenes in each PAI type suggests that the *vap* PAI diversification process is evolutionarily relatively recent. Significantly, the *vap* PAIs are all exquisitely conserved within each plasmid type, with only a few SNPs that do not affect the sequence of the encoded products. This indicates that the plasmid type-specific PAIs are subject to strong selection. The unique association of each *vap* PAI type with a specific animal species suggests that the selective forces involved are host related. The differences between the three *vap* PAIs are mainly accounted for by the *vap* genes, whereas the non-*vap* genes present in the three type-specific PAIs tend to be highly conserved.

Our findings support a two-step model for the evolution of *R. equi* virulence. The process was likely initiated by the acquisition by a saprophytic *Rhodococcus* of a primordial determinant carrying a critical set of *vap* and non-*vap* genes that conferred the ability to survive macrophage attack; this ability is retained as a common feature in the three host-associated *vap* PAI types. This was followed—possibly coinciding with the advent of domestication—by the development of host-tropic properties in response to species-specific selection in farm animals, presumably involving the fast evolution of the *vap* multigene family (Valero-Rello et al. 2015).

## Supplementary Material

Supplementary data are available at *Genome Biology and Evolution* online.

## Supplementary Material

Supplementary Data
