# Supplementary material for: Comparative Genomics of Rhodococcus equi Virulence Plasmids Indicates Host-Driven Evolution of the vap Pathogenicity Island
Source: Genome Biol Evol. 2017 May 1;9(5):1241–7. doi: 10.1093/gbe/evx057 (PMC5434932; doi:10.1093/gbe/evx057)
Supplement: Supplementary Data [file evx057_Supp.pdf]

SUPPLEMENTAL MATERIAL

Supplementary Figures

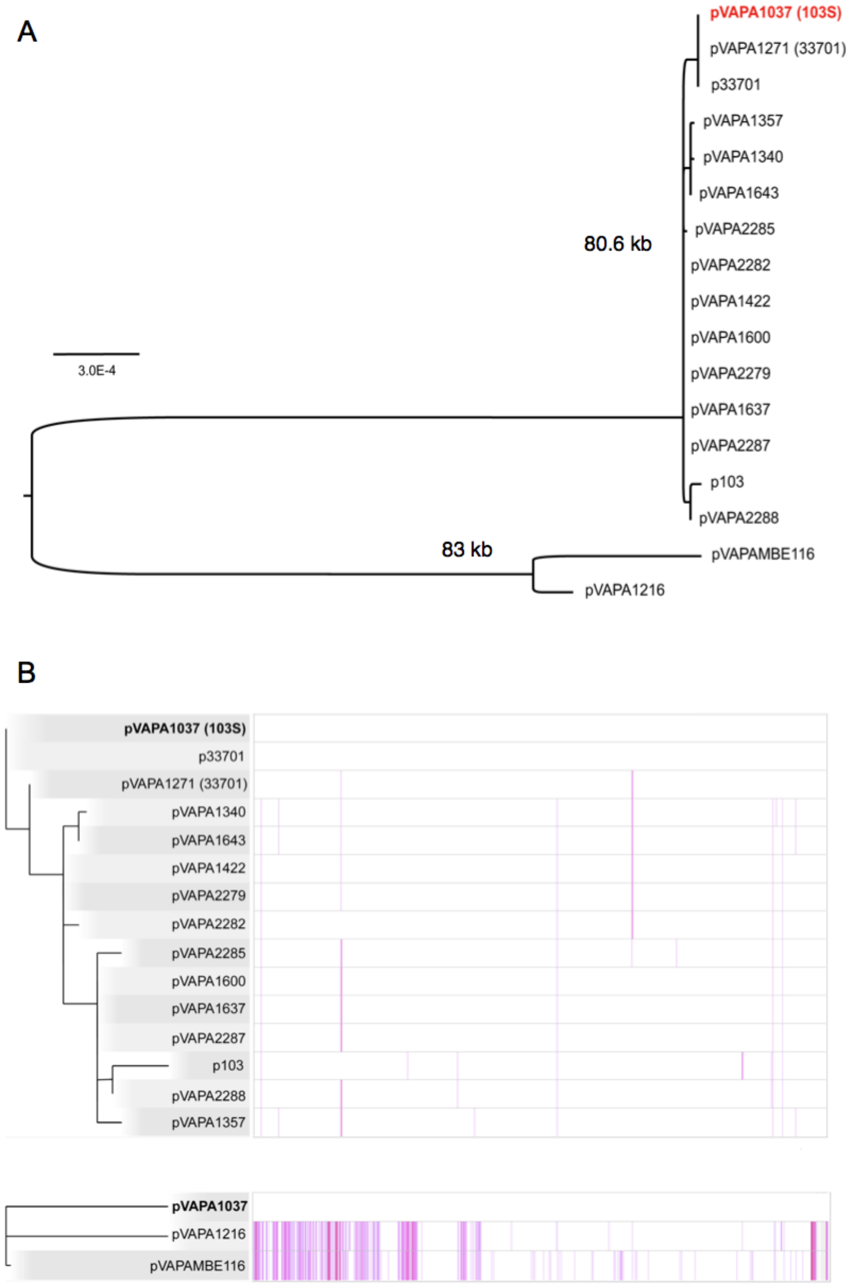

FIG. S1. pVAPA plasmid variability. (*A*) Mid-point rooted phylogenetic tree determined with RealPhy v1.10 (Bertels et al. 2014) using default settings and visualized using FigTree v1.4.2 (http://tree.bio.ed.ac.uk/software/figtree/). Two lineages are clearly identifiable. The predominant 80.6-kb genomic subtype is represented by the reference sequence pVAPA1037 (Letek et al. 2008) (in red), the other lineage corresponds to the 83.1-kb genomic subtype, represented by pVAPA1216 (GenBank accession no. KX443388). The two examples of the 83.1-kb genomic subtype differ from each other by scattered SNPs mostly in intergenic regions, except for three synonymous nucleotide substitutions in PAI genes (one in the *vapI* pseudogene). The 80.6-kb and 83.1-kb pVAPA genomic subtypes correspond to the equine “85-kb” and “87-kb” RFLP plasmid subtypes (Duquesne et al. 2010). (*B*) SNP map of the 80.6-kb (top) and 83.1-kb (bottom) genomic subtypes using pVAPA1037 as reference sequence. Built with Parsnp v1.2 using default settings and visualized with Gingr v1.2 (Treangen et al. 2014).

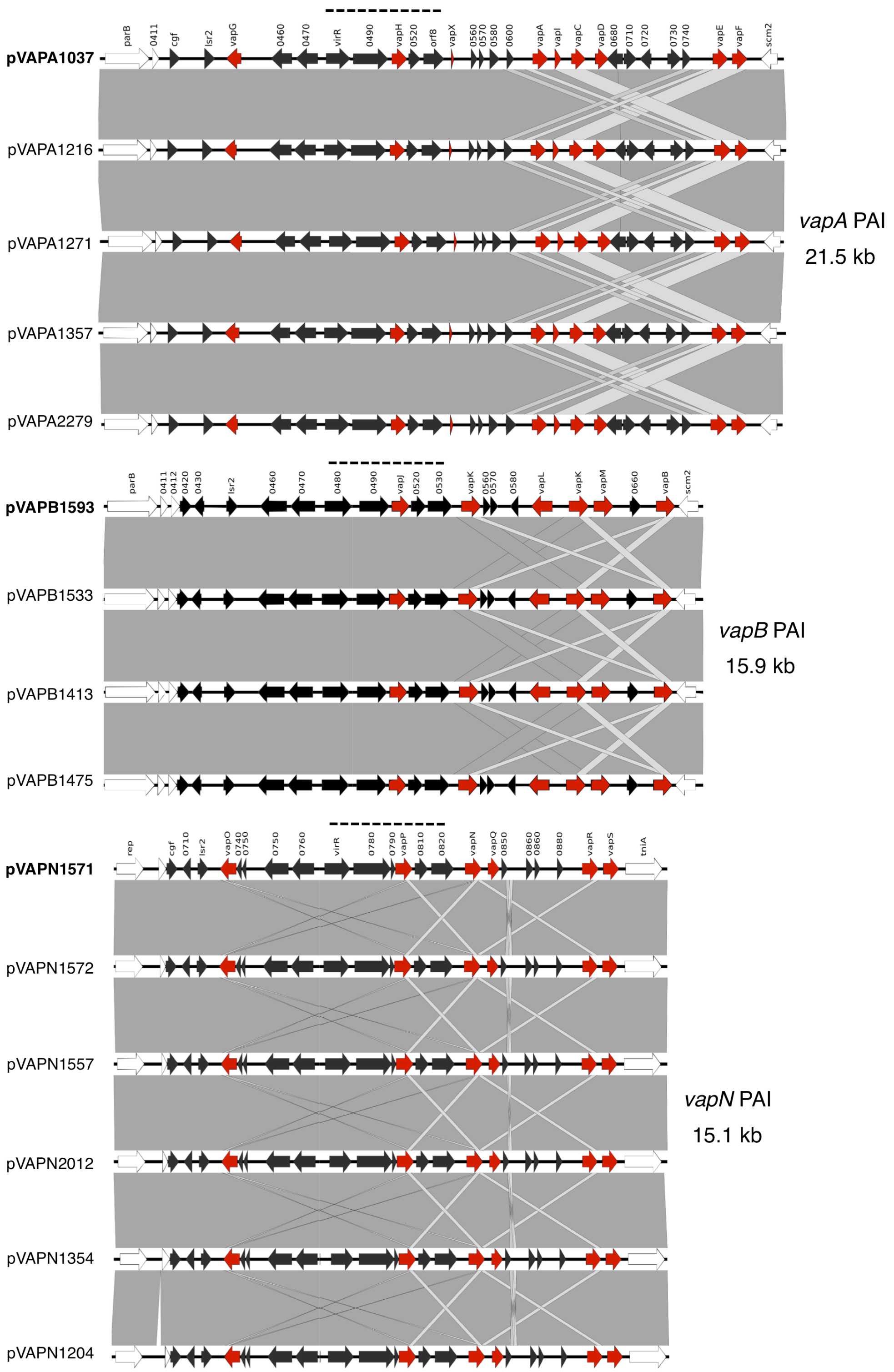

Fig. S2. Comparison of *vapA*, *vapB* and *vapN* PAIs. Representative *vapA* PAIs, including examples from the two pVAPA plasmid genomic subtypes, and all sequenced *vapB* and *vapN* PAIs are shown. Blastn sequence alignments with default settings visualized with Easyfig v2.1 (Sullivan et al. 2011), predominant dark grey stripes indicate >98% sequence identity. Alignments have been centered to the conserved *vir* operon (indicated with a dotted line). PAI genes are in black, *vap* genes in red, non-PAI genes in white. Gene names abbreviated with pVAPX suffix omitted. Reference plasmids of each host-associated type are in bold; see legend to fig. 1 and table S1 for accession nos. and Letek et al. 2008 and Valero-Rello et al. 2015 for a detailed description of the plasmids and corresponding *vap* PAIs. See table S2 for sequence similarity data of the encoded products across the three PAI types. The deviant pVAPN1572 plasmid has a perfectly conserved *vapN* PAI.

Supplementary Text

Comparison of the pVAPA virulence plasmid sequences of strains 103S and ATCC33701 (PAM1271) with those determined by Takai et al. 2000.

When compared to the reference (manually verified) sequence of pVAPA1037 from the genome strain 103S (*) (GenBank accession no. AM947677), the original plasmid sequence of strain 103 (p103 as designated by Takai et al. 2000; GenBank accession no. AF116907) differs by nine SNPs and five short (1-4 bp) indels (fig. S1). However, the pVAPA1037 sequence is identical to that of the original ATCC33701 plasmid sequence (p33701 as per Takai et al. 2000; deposited as pREAT701 in GenBank, accession no. AP001204) (fig. S1). A subculture of ATCC33701 was kept in our isolate collection as PAM1271 and was included in the *R. equi* pangenome study (Anastasi et al. 2016). The pVAPA1271 sequence differs from the original p33701 sequence by four SNPs and two 1-nt indels. Some of these changes are also present in other strains sequenced in this study, suggesting they are genuine. Takai et al. (2000) used different sequencing strategies for their two plasmids, regular shotgun and manual gap joining for p33701, a mixed approach including sequencing of restriction fragment libraries or subcloned cosmid inserts and primer walking for p103, more prone to errors. Indeed, in fig. S1, p103 clusters away from pVAPA1037 together with pVAPA2288, derived from a genome assembly of lower quality (182 contigs vs an average of ≈30-40 for the rest of the genomes). Thus, while in some cases the differences between the sequences reported in Takai et al. (2000) and in our study for the same pVAPA plasmids may reflect short-term genetic drift, they appear to be mostly due to sequencing errors (particularly in the case of the p103 sequence AF116907).

* Isolate 103S used to determine the complete, manually curated reference genome sequence of *R. equi* (Letek et al. 2010) is a subculture of J.F. Prescott's strain 103.

Supplementary Tables

Table S1. *R. equi* virulence plasmids analyzed in this study and corresponding chromosome assemblies. *, reference sequences of each plasmid type.

Strain	Origin	Source host	pVAP type	Plasmid	Size (bp)	GC%	Plasmid accession no.	Reference	Chromosome assembly
103S [a]	Canada	Horse	A	**pVAPA1037** *	80,610	64.61	AM947677	Letek et al. 2008, 2010	FN563149.1
PAM 1271 [a] (ATCC 33701)	Canada	Horse	A	pVAPA1271	80,612	64.61	KX443396	This study	LWIC00000000
PAM 1204	Canada	Sheep	N	pVAPN1204	120,731	66.18	KX443398	This study	LWBN00000000
PAM 1216	Mexico	Horse	A	pVAPA1216	83,136	64.62	KX443388	This study	LWHS00000000
PAM 1340	France	Horse	A	pVAPA1340	80,642	64.62	KX443392	This study	LWHT00000000
PAM 1354	Japan	Human	N	pVAPN1354	120,886	66.20	KX443399	This study	LWHU00000000
PAM 1357	France	Horse	A	pVAPA1357	80,612	64.60	KX443389	This study	LWHV00000000
PAM 1413	Hungary	Human	B	pVAPB1413	106,112	64.89	KX443406	This study	LWHW00000000
PAM 1422	Hungary	Horse	A	pVAPA1422	80,610	64.61	KX443390	This study	LWHX00000000
PAM 1475	Hungary	Pig	B	pVAPB1475	79,250	64.70	KX443397	This study	LWHY00000000
PAM 1496	Hungary	Pig	B [b]	plasmidless	-	-	-	Anastasi et al. 2016	LWHZ00000000
PAM 1533	Slovenia	Pig	B	pVAPB1533	106,558	64.92	KX443407	This study	LWIA00000000
PAM 1557	Ireland	Bovine	N	pVAPN1557	120,857	66.19	KX443400	This study	LWIB00000000
PAM 1571	Ireland	Bovine	N	**pVAPN1571** *	119,931	66.22	KF439868	This study	LWTO00000000
PAM 1572	Ireland	Bovine	N	pVAPN1572	113,690	65.59	KX443401	This study	LXFI00000000
PAM 1593	Spain	Human	B	**pVAPB1593** *	79,251	64.70	AM947676	This study	LXFH00000000
PAM 1600	Australia	Horse	A	pVAPA1600	80,611	64.60	KX443391	This study	LXFG00000000
PAM 1637	Australia	Horse	A	pVAPA1637	80,621	64.61	KX443393	This study	LWHR00000000
PAM 1643	Netherlands	Horse	A	pVAPA1643	80,608	64.61	KX443394	This study	LWTP00000000
PAM 2012	Germany	Bovine	N	pVAPN2012	120,925	66.20	KP851975	This study	LWTY00000000
PAM 2274	US Kentucky	Horse	-	plasmidless	-	-	-	Anastasi et al. 2016	LWTQ00000000
PAM 2276	US Florida	Horse	A [c]	plasmidless	-	-	-	Anastasi et al. 2016	LWTR00000000
PAM 2279	US Florida	Horse	A	pVAPA2279	80,619	64.61	KX443405	This study	LWTS00000000
PAM 2282	US Kentucky	Horse	A	pVAPA2282	80,604	64.61	KX443395	This study	LWTT00000000
PAM 2285	US Florida	Horse	A	pVAPA2285	80,625	64.61	KX443402	This study	LWTU00000000
PAM 2287	US Kentucky	Horse	A	pVAPA2287	80,730	64.61	KX443404	This study	LWTV00000000
PAM 2288	US New York	Horse	A	pVAPA2288	80,754	64.59	KX443403	This study	LWTW00000000
MBE116	France	Horse	A	pVAPAMBE116	83,100	64.60	HM114217	Duquesne et al. 2010	HM114217
DSM20307[T]	Sweden	Horse	-	plasmidless	-	-	-	Anastasi et al. 2016	LWTX00000000
ATCC33707	USA	Human	-	plasmidless	-	-	-	Anastasi et al. 2016	GCA_000164155.2

[a] Subcultures of strains 103 and ATCC 33701, corresponding plasmids previously sequenced by Takai et al. 2000 (see Supplementary Text for details).

[b] Strain was pVAPB positive by PCR when deposited in our collection. [c] Plasmid sequence not complete in the genome assembly and not included in the analyses.

Table S2. Percent sequence identity between type-specific PAI products determined by BLASTp. pVAPA1037 sequences are used as reference. * indicates a pseudogene product. *vir* operon products shaded in gray. See Letek et al. 2008 and Valero-Rello et al. 2015 for details of *vap* allelic variants and non-*vap* genes. All gene products are 100% conserved within each host-associated *vap* PAI type. (*A*) Vap proteins. Amino acid identity of Vap allelic variants relative to pVAPA-encoded Vap proteins (except for VapM). (*B*) Conserved non-Vap PAI products. There are additional small ORFs encoding hypothetical proteins that are only present in one or two of the virulence plasmid types.

A

	(common ancestor *vap* genes) host-associated PAI type-specific Vap allelic variants						
**Plasmid type (ref. seq.)**	**(*vap3*) VapG-L-O**	**(*vap7*) VapH-J-P**	**(*vap1*) VapA-K1,K2/B-N**	**(*vap6*) VapM-Q***	**(*vap2*) VapE/I*-R**	**(*vap5*) VapC/F*-S**	**(*vap4*) VapD**
**pVAPA1037**	100	100	100	allele lost	100<br>47 (I*)	100<br>69 (F*)	100
pVAPB1593	69 (L)	67 (J)	54 (K1,K2)<br>76 (B)	100	allele lost	allele lost	allele lost
pVAPN1571	77 (O)	75 (P)	56 (N)	48 (Q*)	81 (R)	79 (S)	allele lost

B

Non-*vap* gene products

**Plasmid type (ref. seq.)**	**Cgf**	**Lsr2**	**0460**	**0470**	**VirR (Orf4)**	**0490 (IcgA)**	**0520**	**0530 (Orf8)**	**VcgB (Orf10)**
**pVAPA1037**	100	100	100*	100	100	100	100	100	100
pVAPB1593	98	87	76	71	92	80	89	87	66
pVAPN1571	97	86	79	78	95	84	93	88	74

Supplementary Literature Cited

Anastasi E, MacArthur I, Scortti M, Alvarez S, Giguère S, Vázquez-Boland JA. 2016. Pangenome and phylogenomic analysis of the pathogenic actinobacterium *Rhodococcus equi*. Genome Biol Evol 8:3140-3148.

Bertels F, Silander OK, Pachkov M, Rainey PB, van Nimwegen E. 2014. Automated reconstruction of whole-genome phylogenies from short sequence reads. Mol Biol Evol. 31:1077-1088.

Duquesne F, et al. 2010. Analysis of plasmid diversity in 96 *Rhodococcus equi* strains isolated in Normandy (France) and sequencing of the 87-kb type I virulence plasmid. FEMS Microbiol Lett 311:76-81.

Letek M, et al. 2008. Evolution of the *Rhodococcus equi vap* pathogenicity island seen through comparison of host-associated *vapA* and *vapB* virulence plasmids. J Bacteriol 190:5797-5805.

Letek M, et al. 2010. The genome of a pathogenic *Rhodococcus*: cooptive virulence underpinned by key gene acquisitions. PLoS Genet 6:e1001145.

Sullivan MJ, Petty NK, Beatson SA. 2011. Easyfig: a genome comparison visualizer. Bioinformatics 27:1009-1010.

Takai S, et al. 2000. DNA sequence and comparison of virulence plasmids from *Rhodococcus equi* ATCC 33701 and 103. Infect Immun 68:6840–6847.

Treangen TJ, Ondov BD, Koren S, Phillippy AM. 2014. The Harvest suite for rapid core-genome alignment and visualization of thousands of intraspecific microbial genomes. Genome Biol 15:524.

Valero-Rello A, et al. 2015. An invertron-like linear plasmid mediates intracellular survival and virulence in bovine isolates of *Rhodococcus equi*. Infect Immun 83:2725-2737.
